# Case of Superficial Cancer Located at the Pharyngoesophageal Junction Which Was Dissected by Endoscopic Laryngopharyngeal Surgery Combined with Endoscopic Submucosal Dissection

**DOI:** 10.1155/2017/1341059

**Published:** 2017-01-05

**Authors:** Kenro Kawada, Tatsuyuki Kawano, Taro Sugimoto, Kazuya Yamaguchi, Yuudai Kawamura, Toshihiro Matsui, Masafumi Okuda, Taichi Ogo, Yuuichiro Kume, Yutaka Nakajima, Andres Mora, Takuya Okada, Akihiro Hoshino, Yutaka Tokairin, Yasuaki Nakajima, Ryuhei Okada, Yusuke Kiyokawa, Fuminori Nomura, Takahiro Asakage, Ryo Shimoda, Takashi Ito

**Affiliations:** ^1^Department of Gastrointestinal Surgery, Tokyo Medical and Dental University, Tokyo, Japan; ^2^Department of Head and Neck Surgery, Tokyo Medical and Dental University, Tokyo, Japan; ^3^Department of Otorhinolaryngology, Tokyo Medical and Dental University, Tokyo, Japan; ^4^Department of Internal Medicine and Gastrointestinal Endoscopy, Saga Medical School, Saga, Japan; ^5^Department of Human Pathology, Tokyo Medical and Dental University, Tokyo, Japan

## Abstract

*Aims*. In order to determine the indications of transoral surgery for a tumor located at the pharyngoesophageal junction, the trumpet maneuver with transnasal endoscopy was used. Its efficacy is reported here.* Material and Methods*. An 88-year-old woman complaining of dysphagia, diagnosed with cervical esophageal cancer, and hoping to preserve her voice and swallowing function was admitted to our hospital. Conventional endoscopy showed that the tumor had invaded the hypopharynx. When inspecting the hypopharynx and the orifice of the esophagus, we asked the patient to blow hard and puff her cheeks with her mouth closed (trumpet maneuver). After the trumpet maneuver, the pharyngeal mucosa was stretched out. The pedicle of the tumor arose from the left-anterior wall of the pharyngoesophageal junction, so we decided to perform endoscopic resection.* Result*. Under general anesthesia, the curved laryngoscope made it possible to view the whole hypopharynx, including the apex of the piriform sinus and the orifice of the esophagus. The cervical esophageal cancer was pulled up to the hypopharynx. Under collaboration between a head and neck surgeon and an endoscopist, the tumor was resected en bloc by endoscopic laryngopharyngeal surgery combined with endoscopic submucosal dissection.* Conclusion*. Transnasal endoscopy using the trumpet maneuver is useful for a precise diagnosis of the pharyngoesophageal junction. Close collaboration between head and neck surgeons and endoscopists can provide good results in treating tumors of the pharyngoesophageal junction.

## 1. Introduction

With increasing progress in endoscopy, superficial cancers in the head and neck regions are being identified with greater incidence [1–3]. Several methods have been reported as low-invasive transoral surgical approaches, including transoral laser microsurgery (TLM) [4], transoral robotic surgery (TORS) [5], endoscopic laryngopharyngeal surgery (ELPS) [6], and transoral videolaryngoscopic surgery (TOVS) [7]. Endoscopic submucosal dissection (ESD), which was developed as a treatment for gastrointestinal mucosal neoplasia, is now widespread in Japan, and its indications have been expanded to the treatment of pharyngeal lesions [8, 9]. ESD is recognized as an organ preservation strategy, especially with respect to functional outcomes such as swallowing and use of the voice. It is very important to determine the indication, so we must estimate the depth of tumor invasion precisely at the preoperative examination. However, circumferential observation of the hypopharyngeal mucosa is difficult during conventional endoscopy due to its anatomically closed nature. We previously reported the utility of transnasal endoscopy using the trumpet maneuver for precise inspection before treatment [10]. We herein report a case of a tumor located at the pharyngoesophageal junction treated by ELPS combined with ESD.

## 2. Case Presentation

**Figure 1:**
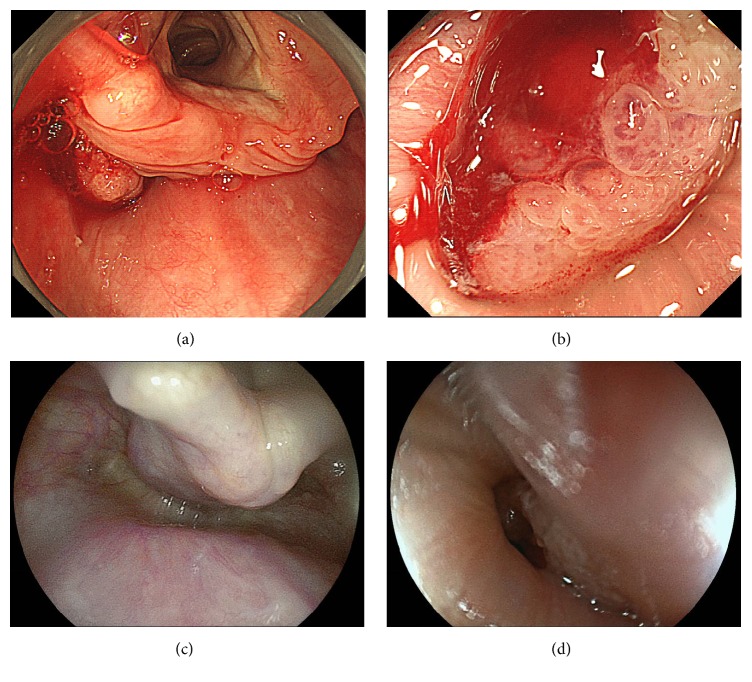
(a) Conventional endoscopy showed that the protruded mass was located at the left piriform sinus. (b) The tumor was very movable and was diagnosed as cervical esophageal cancer. (c) Transnasal endoscopy using the trumpet maneuver showed that the tumor had not widely invaded the hypopharynx. (d) The pedicle of the tumor was located at the left-anterior wall of the pharyngoesophageal junction.

**Figure 2:**
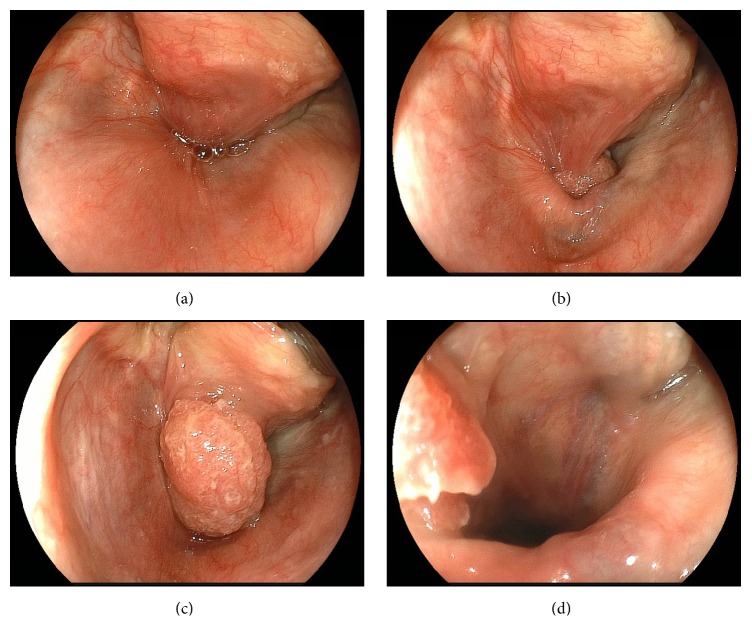
(a) The curved laryngoscope enabled a view of the whole hypopharynx, including the apex of the piriform sinus and the orifice of the esophagus. The tumor was invisible from the hypopharynx. (b) The tumor was pulled up from the cervical esophagus to the hypopharynx. (c) The pedicle of the tumor was mainly located at the postcricoid area. (d) The flat, superficial part and the anal side of the tumor had spread to the cervical esophagus.

An 88-year-old woman had suffered from a cough for the past 2 years and had recently felt dysphagia. The dysphagia progressed to the extent that she was unable to tolerate solid foods. She also reported an episode of regurgitation of a mass into her mouth. Three months before, she was diagnosed with cervical esophageal cancer at the clinic near her home, at which point she was admitted to a local tertiary medical center. On the further examination, she was diagnosed with cervical esophageal cancer invading the hypopharynx (Figures 1(a) and 1(b)). A histopathological examination revealed squamous cell carcinoma. Computed tomography and positron emission tomography (PET) revealed no lymph nodal metastasis or distant metastasis. She was recommended to undergo total pharyngolaryngoesophagectomy or chemoradiotherapy. However, she was elderly; she refused these invasive treatments and hoped to preserve her voice and swallowing function. She was therefore referred to our hospital for a second opinion. The present patient received a transnasal endoscopic examination. We have routinely performed the trumpet maneuver using transnasal endoscopy for patients with esophageal cancer since 2009, using the following procedure. First, the patient is asked to bow the head deeply in the left lateral position. We then place a hand on the back of the patient's head and push it forward. To examine the hypopharynx and the orifice of the esophagus, the patient is asked to blow hard and puff the cheeks while the mouth remains closed (trumpet maneuver). The image of the pharynx using the trumpet maneuver with transnasal endoscopy in the present patient is shown in Figure 1(c). The postcricoid was difficult to visualize during esophagogastroduodenoscopy; however, the view could be improved by using trumpet maneuver. We therefore concluded that the tumor of the cervical esophagus had not invaded the hypopharynx. The pedicle of the tumor was located at the left-anterior wall of the pharyngoesophageal junction (Figure 1(d)). We therefore planned to observe it under general anesthesia. A specially designed curved laryngoscope was inserted into the anesthetized patient to create a working space in the pharyngoesophageal lumen. Initially, the tumor was invisible from the hypopharynx (Figure 2(a)), as most of it was located in the cervical esophagus. Using the forceps, the tumor was pulled up from the cervical esophagus to the hypopharynx (Figure 2(b)). The pedicle of the tumor was mainly located at the postcricoid area (Figure 2(c)). The flat superficial part and anal side of the tumor had spread to the cervical esophagus (Figure 2(b)). It was easy to grasp the pedicle with the forceps inserted transorally, and the lesion was moving well, so we concluded that the tumor had not invaded the muscle layer.

## 3. Therapeutic Procedure

**Figure 3:**
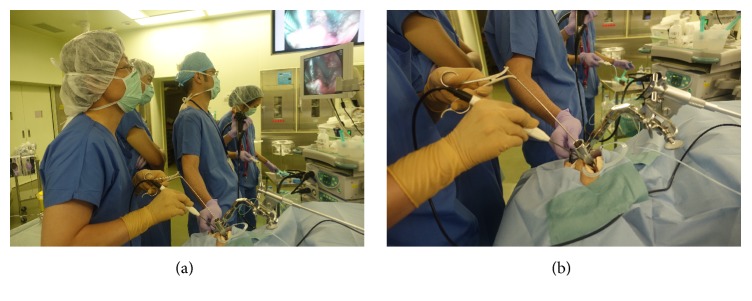
(a) ELPS is a video-assisted surgery performed by two physicians: a head and neck surgeon acting as the operator and an endoscopic specialist acting as the endoscopist. They observe the same TV monitor in the operation room. (b) The operator uses the curved forceps and the electric needle knife, while the endoscopist inserts the transnasal endoscope through the nose.

Endoscopic laryngopharyngeal surgery is a video-assisted surgery performed by two physicians (Figure 3(a)): an operator and endoscopist who is an endoscopic specialist [7]. The endoscopist inserts the esophagogastroduodenoscope and obtains images of the lesion on a monitor. The operator uses the curved forceps and the electric needle knife, while the endoscopist inserts the transnasal endoscope through the nose (Figure 3(b)).

**Figure 4:**
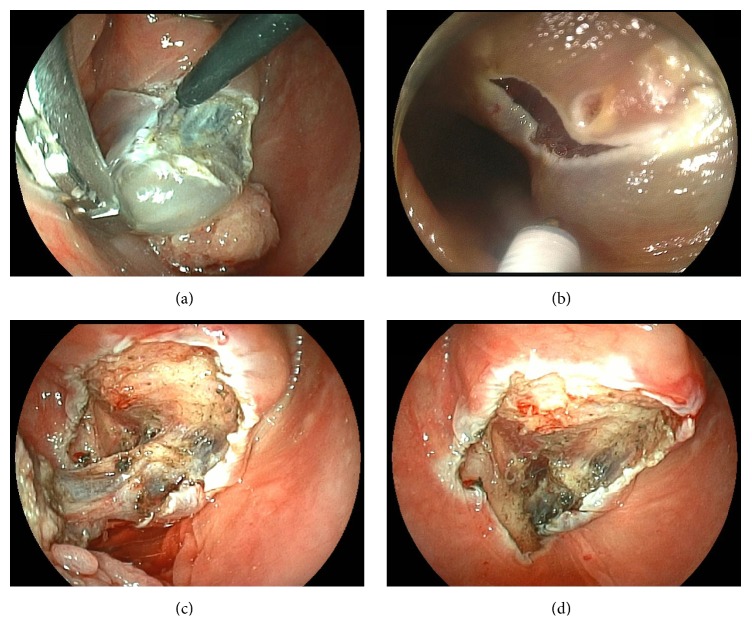
(a) After saline was injected into the subepithelial layer, the head and neck surgeon dissected the lesion using the orally inserted curved grasping forceps with one hand and the orally inserted curved electric needle knife with the other. (b) The esophageal portion was resected by ESD. (c) The pharyngeal portion was resected by ELPS. (d) After tumor removal, the tumor was resected en bloc.

In the present patient, the endoscope first identified and demarcated the lesion margins using iodine dye, which was then marked by endoscopist. Next, a solution of epinephrine (0.02 mg/mL) and saline was then injected into the subepithelial layer to separate the mucosa from the muscle layer proper. Then a head and neck surgeon performed the circumferential mucosal incision using an electric needle knife (KD-600, Olympus Medical Systems, Tokyo, Japan) and forceps inserted transorally (Figure 4(a)). At the esophageal portion, the needle knife could not reach the lesion, inserted transorally as it was, so it was difficult for the head and neck surgeon to perform the operation alone. An endoscopist was therefore called in to help the operator dissect the anal margin using the electric knife inserted through a flexible endoscope (Figure 4(b), ESD procedure). After the circumferential incision, dissection was performed using the orally inserted curved grasping forceps with an electric knife in combination (Figure 4(c) with video https://www.youtube.com/watch?v=2FCFVLQtAN8). The tumor was resected en bloc (Figure 4(d)). Finally, triamcinolone was injected endoscopically to prevent the posttreatment formation of stricture.

The operation time was 62 minutes. The patient was extubated immediately after surgery. The fasting period was two days after surgery, and the postoperative hospital stay was five days. There were no complications and no hoarseness or swallowing discomfort.

**Figure 5:**
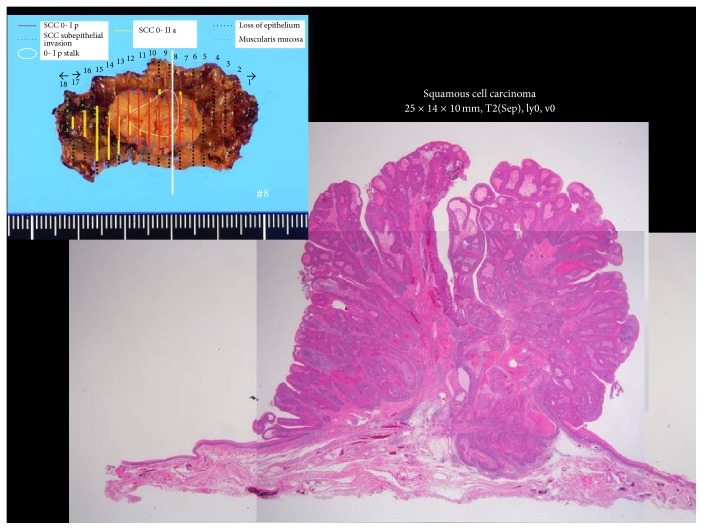
A histopathological examination revealed a diagnosis of invasive squamous cell carcinoma. A 25 × 18 × 10 mm superficial lesion was removed by en bloc resection.

**Figure 6:**
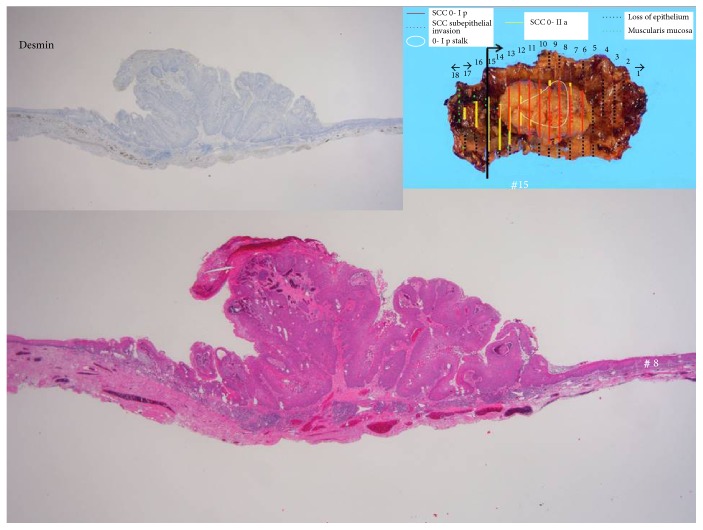
At the anal side of the specimen, the muscularis mucosa was examined, and the tumor was confirmed to have spread to the cervical esophagus.

**Figure 7:**
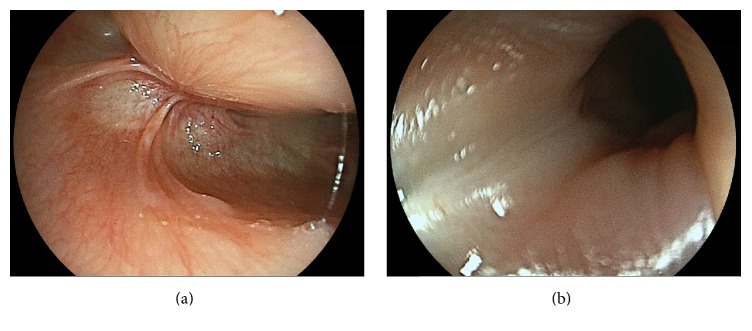
(a) At 8 months after treatment, no local recurrence was observed. (b) No stricture was observed at the cervical esophagus.

The histopathological findings were squamous cell carcinoma, 25 × 18 × 10 mm, subepithelial invasion, no microvascular permeation, and a negative vertical margin (Figure 5). On the anal side of the specimen, the muscularis mucosa was examined (Figure 6), and the tumor was confirmed to have spread to the cervical esophagus. According to the TNM classification, the final stage was Stage III (T3N0M0). The follow-up examinations after treatment included cervical ultrasound and the measurement of her tumor marker levels every three months and computed tomography every six months. Balloon dilation was not required. The patient is currently alive with no recurrence at 8 months after the surgery (Figure 7(a)), and there is no stricture at the cervical esophagus (Figure 7(b)).

## 4. Discussion

With increasing progress in esophagogastroduodenoscopy, superficial cancers in the head and neck regions are being identified with greater incidence. Transoral surgery is becoming a major strategy in the treatment of laryngopharyngeal cancer [11]. However, the choice of an adequate therapeutic strategy for treating neoplasms located at the pharyngoesophageal junction is not clearly defined and therefore difficult. Chemoradiotherapy is widely performed at present but is associated with a high frequency of complications during and after treatment [12]. Total pharyngolaryngoesophagectomy is considered the most complicated and most invasive surgery for the gastrointestinal tract [13]. In determining the treatment strategy, the stage and location of the tumor and the general status of the patient should be considered. The indications of voice-preserving surgery can be examined using transoral endoscopy or esophagogram, but it can sometimes be difficult to observe the pharyngoesophageal junction. However, determining the precise location of tumor is important in determining the optimum method of treatment. We previously reported the utility of transnasal endoscopy using the trumpet maneuver for observing the pharyngoesophageal junction [14]. This method is also useful for determining the oral surgical margin of cervical esophageal cancer preoperatively [15].

We first diagnosed the patient with cervical esophageal cancer that had not widely invaded the hypopharynx and the pedicle was located only in the junction. However, the tumor was actually mainly located at the postcricoid, and a curved laryngoscope under general anesthesia was very effective in obtaining a wide space to examine the hypopharynx. This preoperative study using transnasal endoscopy was also important for determining whether or not her larynx could be preserved.

Kawano et al. [16] previously reported a case of superficial carcinoma located at the pharyngoesophageal junction treated with endoscopic mucosal resection (EMR). In EMR, a small-diameter snare (SD-7P, Olympus, Tokyo, Japan) is used for resection. A weak point of EMR is that endoscopists cannot confirm an accurate cutting line until resection is performed, occasionally requiring multiple instances of resection [17]. ESD is now a standard procedure in the gastrointestinal tract in Japan, with a gastroenterologist easily resecting the mucosa containing the lesion at the submucosal level of the esophageal region. We performed ELPS combining with ESD in the present patient.

In Japan, most cases of superficial squamous cell carcinoma located at the cervical esophagus are treated endoscopically. To avoid the perforation of esophageal wall, saline is first injected into the subepithelial layer to separate the mucosa from the muscle layer proper. It was reported that the endoscopic injection of steroid is useful for preventing stricture after esophageal ESD [18]. In this case, we could easily perform surgery without any complications. Collaboration between a head and neck surgeon and a gastroenterologist on ELPS combined with ESD has been reported useful for treating even the hypopharyngeal lesions that have spread to the cervical esophagus which is difficult with TLM, TOVS, and TORS [19]. Although we do not have any robotic surgery systems in our institution, we were able to perform the treatment with human power alone.

More cases and longer follow-up periods will be required to obtain conclusive findings, and future studies will need to determine the indications of this treatment.

## 5. Conclusion

Transnasal endoscopy using the trumpet maneuver is useful for diagnosing the pharyngoesophageal junction. Collaboration between head and neck surgeon and an endoscopist can provide a good result in treating tumors of the pharyngoesophageal junction. ELPS combined with ESD for treating superficial neoplasm of the pharyngoesophageal junction is a promising therapeutic option for elderly patients.
